# Modeling Posttreatment Prognosis of Skin Lesions in Patients With Psoriasis in China

**DOI:** 10.1001/jamanetworkopen.2023.6795

**Published:** 2023-04-06

**Authors:** Zhihui Yang, Shasha Han, Peng Wu, Mingyue Wang, Ruoyu Li, Xiao-Hua Zhou, Hang Li

**Affiliations:** 1 Department of Dermatology and Venerology, Peking University First Hospital, Beijing, China; 2 Beijing Key Laboratory of Molecular Diagnosis of Dermatoses, Peking University First Hospital, Beijing, China; 3 National Clinical Research Center for Skin and Immune Diseases, Beijing, China; 4 NMPA Key Laboratory for Quality Control and Evaluation of Cosmetics, Peking University First Hospital, Beijing, China; 5 School of Population Medicine and Public Health, Chinese Academy of Medical Sciences & Peking Union Medical College, Beijing, China; 6 Beijing International Center for Mathematical Research, Peking University, Beijing, China; 7 School of Mathematics and Statistics, Beijing Technology and Business University, Beijing, China; 8 Research Center for Consumption Big Data and Intelligent Decision-Making, Beijing Technology and Business University, Beijing, China; 9 Department of Biostatistics, School of Public Health, Peking University, Beijing, China; 10 National Engineering Laboratory of Big Data Analysis and Applied Technology, Peking University, Beijing, China; 11 Peking University–Yunnan Baiyao International Medical Research Center, Beijing, China

## Abstract

**Importance:**

Understanding the posttreatment prognosis of skin lesions in patients with psoriasis is essential for improving patients’ treatment satisfaction.

**Objectives:**

To model the prognosis of skin lesions for patients with psoriasis after 3 types of therapy.

**Design, Setting, and Participants:**

This prospective cohort study included patients with psoriasis who visited a dermatologist and were enrolled in the platform of the Psoriasis Standardized Diagnosis and Treatment Center in China from August 2020 to December 2021.

**Interventions:**

Biologic, traditional, and systemic therapy for psoriasis.

**Main Outcomes and Measures:**

Skin lesions were measured by the Investigator’s Global Assessment (IGA) scale subsumed into 4 stages of severity (IGA 0/1, IGA 2, IGA 3, and IGA 4), with higher scores indicating higher severity. The matching method was used to balance baseline covariates between patients receiving each of the 3 treatments. Transition probabilities from IGA scores at baseline to 0 to 1 month and 1 to 12 months were estimated.

**Results:**

A total of 8767 patients were included in the final analysis (median age, 38.6 years [IQR, 28.7-52.8 years]; 5809 [66.3%] male). Across the 3 therapies, as the follow-up duration increased, the probability of improvement transition into a less severe IGA stage (from IGA 4 to IGA 0/1) increased from 0.19 (95% CI, 0.18-0.21) in 0 to 1 month to 0.36 (95% CI, 0.34-0.37) in 1 to 12 months. Biologic therapy was associated with greater improvement transitions for severe conditions, with transition probabilities from IGA 4 to IGA 0/1 increasing by 0.06 (95% CI, 0.02-0.09) vs traditional therapy and by 0.06 (95% CI, 0.03-0.09) vs systemic therapy in 0 to 1 month and by 0.08 (95% CI, 0.04-0.12) vs traditional therapy and 0.11 (95% CI, 0.07-0.14) vs systemic therapy in 1 to 12 months.

**Conclusions and Relevance:**

This cohort study modeling psoriasis prognosis provided a complete prognosis of skin lesions, and biologic therapy was associated with improved prognosis of moderate to severe psoriasis compared with traditional and systemic therapies. The study provides insight on using transition diagrams to assess psoriasis prognosis and to communicate with patients in clinical practice.

## Key Points

**Question:**

What is the prognosis of skin lesions measured by transition diagrams in patients with psoriasis after 3 types of therapy—biologic, traditional, and systemic?

**Findings:**

In this cohort study modeling psoriasis prognosis among 8767 patients in China, biologic therapy was associated with improved prognosis for moderate to severe psoriasis compared with traditional and systemic therapies. The transition diagram was demonstrated to be a useful modeling tool.

**Meaning:**

The findings suggest that biologic therapy should be considered for the treatment of skin lesions in patients with moderate to severe psoriasis and that transition diagrams may be considered for improving communications between clinicians and patients.

## Teaser

This cohort study uses transition diagrams to estimate the prognosis of skin lesions after biologic, traditional, and systemic therapies among patients with psoriasis in China.

## Introduction

Psoriasis is a common chronic inflammatory disease occurring worldwide.^1,2,3^ The condition is characterized by skin plaques with irregular borders and silvery scales, which can greatly affect patients’ quality of life^4^ and have been associated with a higher risk of developing depression and anxiety or committing suicide.^5,6,7^ Quick skin healing and improvement are often given the highest priority by patients.^8^ However, due to the relapsing nature of psoriasis and the need for long-time treatment adherence, it is challenging to communicate efficiently with patients in managing psoriasis.^9^ Nontreatment, undertreatment, and low treatment adherence remain significant problems worldwide. In China, guidelines for psoriasis recommend starting with topical drugs for patients with mild psoriasis and progressing to more potent therapies (including phototherapy and systemic therapy) for severe conditions.^9^ However, phototherapy is time consuming, while biologic therapy is costly. As such, patients with psoriasis are often left with nonbiologic systemic medicines or even discouraged from seeing physicians. A better understanding of the posttreatment prognosis of skin lesions is essential to improve communications between patients and clinicians.^10,11,12^

Prognoses of skin lesions are conventionally assessed by the Psoriasis Area and Severity Index (PASI), a dermatology-specific quality-of-life measure (the Dermatology Life Quality Index [DLQI]),^13,14,15,16,17^ and the presence of comorbidities.^18,19^ However, PASI and DLQI are complicated measures with scales that are difficult to understand by clinicians and patients.^20^ Neither has been routinely adopted in clinical practice in China.^21,22^ Also, the presence of comorbidities cannot capture the treatment needs of patients. Thus, a more easily implementable instrument is needed.

We modeled the posttreatment prognosis of skin lesions using transition diagrams among skin lesion scales. A transition diagram is a widely used tool for assessing disease development in chronic diseases^23,24,25^ and models the probabilities of transitioning among health stages over time.^26^ It is useful for understanding the course of diseases and the chances of getting better.^24^ The instrument has not been used in the prognosis of psoriasis, possibly due to the complexity of skin lesion scales. We used the 5-point Investigator’s Global Assessment (IGA) scale, which is simple and valid for assessing plaque psoriasis severity.^27^ We estimated the transition probabilities in the diagrams with a large cohort of patients in China. Our study had 2 complementary objectives. The first, a clinical objective, was to model the posttreatment prognosis of skin lesions, filling an important clinical knowledge gap for understanding the prognosis of skin lesions after therapy. The second, a methodologic objective, was to ascertain whether transition diagrams can be used to differentiate therapy effectiveness, filling important methodologic needs in improving clinical communications between patients and clinicians.

## Methods

### Study Design

In this cohort study, data were collected from the platform of the Psoriasis Standardized Diagnosis and Treatment Center, led by the National Clinical Research Center for Skin and Immune Diseases and in collaboration with 217 hospitals across 86 cities of 28 provinces in China.^28^ A cohort of patients who visited dermatologists in the collaborating hospitals from August 2020 to December 2021 was enrolled. The study was approved by the Human Genetic Resources Management Office of the Ministry of Science and Technology of China and the ethics committee of Peking University First Hospital. All patients signed informed consent forms at enrollment. Data on demographic and clinical characteristics, skin lesion measures, and treatment decisions were collected at enrollment. Patients’ adherence to treatments, skin lesion measures, and adverse events were collected at follow-up visits. This study followed the Strengthening the Reporting of Observational Studies in Epidemiology (STROBE) reporting guideline.

For the present study, we excluded patients whose treatment information was missing, unclear, or discontinued. Patients treated with Chinese herbs were additionally excluded because there is large uncertainty around the components and dosage of herbs, which are critical to evaluate the effects of herbs.^29,30^ Also, we excluded patients who had been exposed to biologic therapy less than a year prior to enrollment.^31^ Furthermore, because biologic therapy is primarily used for plaque psoriasis in China,^9^ we included only patients who were diagnosed with plaque psoriasis and whose baseline IGA scores were known.

### Treatment of Psoriasis

We considered 3 treatment conditions: biologic therapy, traditional therapy, and systemic therapy. Biologic therapy represents treatment with biologics, including anti–tumor necrosis factor α agents (eg, adalimumab, etanercept, and infliximab) and interleukin inhibitors (eg, ustekinumab, secukinumab, ixekizumab, and guselkumab). Traditional therapy refers to externally applied therapies, including phototherapy and topical medications such as topical corticosteroids, vitamin D_3_ analogues and their combination agents, retinoids, salicylic acid, calcineurin inhibitors, and benvitimod. Systemic therapy represents nonbiologic systemic medications, including cyclosporine, methotrexate, acitretin, leflunomide, and small-molecule drugs.

### Posttreatment Prognosis of Skin Lesions

The prognosis of skin lesions was assessed by transition probabilities from the current stage of severity into the stage of severity at the follow-up visits in 0 to 1 month and 1 to 12 months separately. Skin lesions were measured by IGA scales. We subsumed the IGA scores into 4 scales of severity (IGA 0/1, IGA 2, IGA 3, and IGA 4) as in clinical practice, with higher scores indicating higher severity. As such, we needed to estimate 4 × 4 = 16 transition probabilities for each of the 3 therapies.

### Statistical Analysis

Due to the potential confounding between the baseline characteristics and the association between treatment and transition probabilities, a direct contrast of transitions among the 3 treatment groups may be biased. To reduce biases, we used the matching method to balance the baseline characteristics among the 3 treatment groups.^32,33^ For each patient in the group receiving biologic therapy, we found 2 matched individuals—1 from the group receiving traditional therapy and the other from the group receiving systemic therapy—by matching with replacement based on Mahalanobis distance. The procedure was repeated for each patient in the group receiving traditional therapy and systemic therapy separately. As such, a total of 8767 pairs were generated.

Variables that were used in the matching included sex, age, employment, marital status, educational level, smoking status, psoriasis duration, family history, lesions on particular body areas, PASI, body surface area affected by psoriasis lesions, IGA scores, DLQI, and comorbidity (eAppendix 1 in Supplement 1).^34^ Variables had missing percentages ranging from 1.5% to 8.1%. Missing values were imputed using the multiple imputation method, with 100 imputed data sets. Matching was done for each imputed data set. Transition probabilities were calculated within each of the matched data sets and were merged using the Rubin rule.^35,36^ Transition probabilities from the baseline IGA scores to IGA scores in 0 to 1 month and 1 to 12 months were estimated separately. Missing IGA scores in the follow-up visits (5443 [62.1%] in 0 to 1 month and 4274 [48.8%] in 1 to 12 months) were imputed using the last-observation-carried-forward method. When IGA scores in the follow-up visits had different values, only the IGA score in the last visit was used in the analysis.

Descriptive analysis was performed using Stata, release 17 (StataCorp LLC). Matching was performed using R, version 4.0.3 (R Foundation for Statistical Computing).

## Results

### Baseline Characteristics Before and After Matching

Of 16 523 patients who visited dermatologists in the collaborating hospitals during the study period, 13 565 patients had at least 1 follow-up visit within a year and 2958 (17.9%) were lost to follow-up. Among the 8767 patients included in the final analysis, the median age was 38.6 years (IQR, 28.7-52.8 years), 2950 (33.6%) were female, and 5809 (66.3%) were male (data on sex were missing for 8 patients). A total of 2860 patients (32.6%) were primarily treated with biologics, 4706 (53.7%) with traditional therapy, and 1201 (13.7%) with systemic medication (eFigure in Supplement 1). Descriptive statistics of the variables before and after matching are presented in Table 1. Before matching, there were substantial differences in baseline characteristics between the group receiving biologic therapy and the groups receiving the other 2 therapies. For example, patients who received biologic therapy vs patients receiving traditional therapy and patients receiving systemic therapy were more likely to have longer psoriasis duration (median of 10 years [IQR, 4-18 years] vs 4 years [IQR, 1-11 years] vs 6 years [IQR, 2-14 years]), higher median PASI score (14.3 [IQR, 7.5-21.6] vs 5.8 [IQR, 2.7-12.0] vs 10.8 [IQR, 5.6-18.3]), higher median DLQI score (10 [IQR, 5-16] vs 6 [IQR, 2-10] vs 9 [IQR, 4-13]), a family history of psoriasis (529 [18.5%] vs 712 [15.1%] vs 159 [13.2%]), and particular area involvement (nail: 924 [32.3%] vs 801 [17.0%] vs 346 [28.8%]; scalp: 2086 [72.9%] vs 2977 [63.3%] vs 775 [64.5%]; palmoplantar: 781 [27.3%] vs 629 [13.4%] vs 284 [23.6%]; genital: 546 [19.1%] vs 454 [9.6%] vs 156 [13.0%]). After matching, the baseline characteristics among the 3 groups were well balanced by hypothesis testing (eAppendix 2 and eTable in Supplement 1) and covariates in the 3 groups were more similar (Table 1).

**Table 1.** Patient Characteristics by Treatment Group at Enrollment, 2020-2021^a^

Characteristic	Primary cohort, No. (%)			Matched cohort, % (95% CI)^b^		
	Traditional therapy (n = 4706)	Systemic therapy (n = 1201)	Biologic therapy (n = 2860)	Traditional therapy	Systemic therapy	Biologic therapy
Sex						
Female	1720 (36.5)	343 (28.6)	887 (31.0)	33.9 (33.5-34.3)	29.3 (28.6-30.0)	31.2 (30.7-31.7)
Male	2983 (63.4)	856 (71.3)	1970 (68.9)	66.1 (65.7-66.5)	70.7 (70.0-71.4)	68.8 (68.3-69.3)
Age, median (IQR or 95% CI), y	36.7 (26.4-52.1)	46.7 (32.8-57.6)	38.9 (30.4-51.7)	38.4 (38.4-38.5)	38.9 (38.8-39.0)	38.5 (38.4-38.6)
BMI, median (IQR or 95% CI)	23.7 (21.3-26.2)	24.2 (22.1-26.2)	24.2 (22.0-26.7)	24.0 (23.9-24.0)	24.1 (24.0-24.2)	24.2 (24.2-24.2)
Marital status						
Married	3325 (70.7)	964 (80.3)	2143 (74.9)	75.6 (75.3-76.0)	75.7 (74.9-76.4)	73.8 (73.4-74.2)
Unmarried	1317 (28.0)	214 (17.8)	670 (23.4)	24.4 (24.0-24.7)	24.3 (23.6-25.1)	26.2 (25.8-26.6)
Employment						
Full time	2598 (55.2)	663 (55.2)	1800 (62.9)	59.0 (58.5-59.5)	61.2 (60.4-62.0)	61.3 (60.8-61.7)
Other^c^	2045 (43.5)	515 (42.9)	1013 (35.4)	41.0 (40.5-41.5)	38.8 (38.0-39.6)	38.7 (38.3-39.2)
Educational level						
College degree or higher	1325 (28.2)	257 (21.4)	1004 (35.1)	28.1 (27.6-28.5)	26.1 (25.3-26.9)	35.1 (34.5-35.7)
High school or lower	3318 (70.5)	921 (76.7)	1809 (62.3)	71.9 (71.5-72.4)	73.9 (73.1-74.7)	64.9 (64.3-65.5)
Smoking status						
Current	1155 (24.5)	338 (28.1)	750 (26.2)	25.5 (25.1-25.9)	26.9 (26.0-27.7)	24.1 (23.6-24.7)
Previous or nonsmoker	3488 (74.1)	840 (69.9)	2063 (72.1)	74.5 (74.1-74.9)	73.1 (72.3-74.0)	75.9 (75.3-76.4)
Psoriasis duration, median (IQR or 95% CI), y	4 (1-11)	6 (2-14)	10 (4-18)	6 (6-6)	5 (5-5)	6 (6-6)
Family history of psoriasis						
Positive	712 (15.1)	159 (13.2)	529 (18.5)	15.6 (15.0-16.1)	13.8 (13.0-14.6)	17.1 (16.4-17.8)
Negative	3504 (74.5)	923 (76.9)	2030 (71.0)	84.4 (83.9-85.0)	86.2 (85.4-87.0)	82.9 (82.2-83.6)
Lesions on particular areas						
Nail						
Affected	801 (17.0)	346 (28.8)	924 (32.3)	19.4 (18.9-19.9)	24.3 (23.6-25.0)	25.9 (25.3-26.5)
Unaffected	3719 (79.0)	820 (68.3)	1820 (63.6)	80.6 (80.1-81.1)	75.7 (75.0-76.4)	74.1 (73.5-74.7)
Scalp						
Affected	2977 (63.3)	775 (64.5)	2086 (72.9)	67.3 (66.8-67.8)	67.2 (66.3-68.1)	69.8 (69.2-70.4)
Unaffected	1653 (35.1)	401 (33.4)	740 (25.9)	32.7 (32.2-33.2)	32.8 (31.9-33.7)	30.2 (29.6-30.8)
Palmoplantar						
Affected	629 (13.4)	284 (23.6)	781 (27.3)	15.4 (15.0-15.8)	17.3 (16.7-18.0)	20.2 (19.7-20.7)
Unaffected	3971 (84.4)	886 (73.8)	2004 (70.1)	84.6 (84.2-85.0)	82.7 (82.0-83.3)	79.8 (79.3-80.3)
Genital						
Affected	454 (9.6)	156 (13.0)	546 (19.1)	10.7 (10.3-11.1)	10.3 (9.7-10.8)	14.1 (13.7-14.6)
Unaffected	4115 (87.4)	1006 (83.8)	2217 (77.5)	89.3 (88.9-89.7)	89.7 (89.2-90.3)	85.9 (85.4-86.3)
Disease severity, median (IQR or 95% CI)						
PASI	5.8 (2.7-12.0)	10.8 (5.6-18.3)	14.3 (7.5-21.6)	8.8 (8.7-8.9)	9.0 (9.0-9.0)	9.0 (9.0-9.0)
BSA affected by psoriasis lesions, %	8.0 (3.0-20.0)	20.0 (8.0-40.0)	21.0 (10.0-40.0)	14.0 (13.3-14.7)	14.4 (14.0-14.8)	14.0 (13.3-14.7)
DLQI	6 (2-10)	9 (4-13)	10 (5-16)	8 (8-8)	8 (8-8)	8 (8-8)
Comorbidities						
Positive	653 (13.9)	236 (19.7)	431 (15.1)	15.3 (14.8-15.8)	15.9 (15.1-16.7)	15.0 (14.4-15.6)
Negative	3682 (78.2)	871 (72.5)	2186 (76.4)	84.7 (84.2-85.2)	84.1 (83.3-84.9)	85.0 (84.4-85.6)

Abbreviations: BMI, body mass index (calculated as weight in kilograms divided by height in meters squared); BSA, body surface area; DLQI, Dermatology Life Quality Index; PASI, Psoriasis Area and Severity Index.

^a^ Patients whose characteristics were missing were not counted; thus, the numbers may not sum to group total counts or to 100%.

^b^ Data were calculated from 20 multiple imputed data sets.

^c^ Other included part-time workers, students, retired participants, and unemployed participants.

### Posttreatment Prognoses in 0 to 1 Month and in 1 to 12 Months

**Figure 1.**
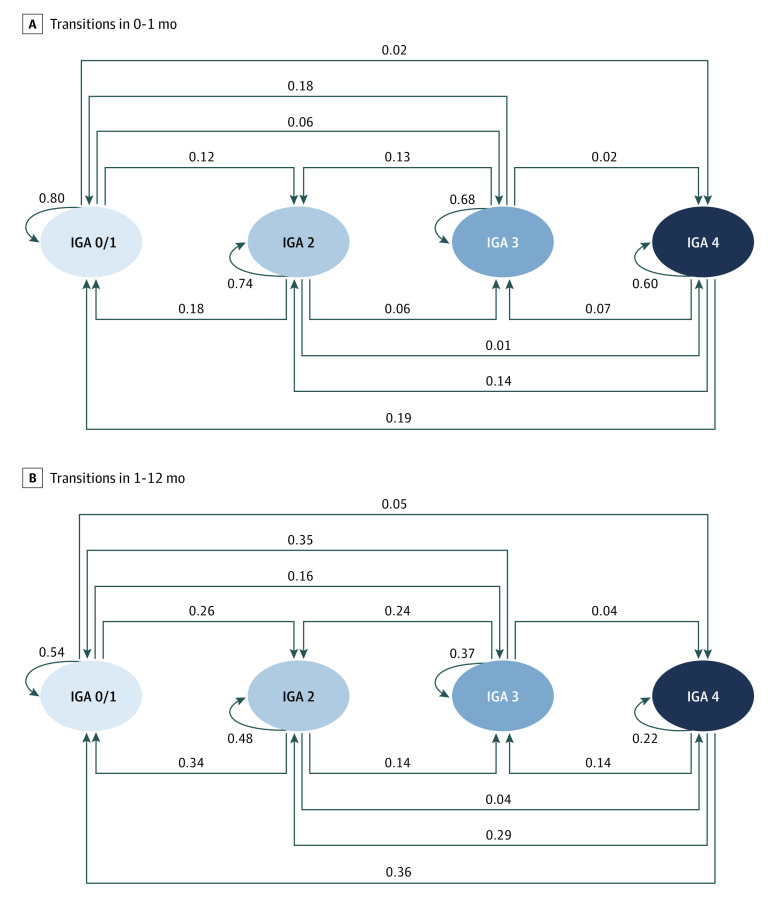
Overall Posttreatment Transition Diagrams Among Investigator’s Global Assessment (IGA) Stages Across 3 Therapies Data shown are probabilities of transitioning from the baseline stage to posttreatment stage in 0 to 1 months (A) and 1 to 12 months (B).

The transition diagrams of skin lesions across all therapies are shown in Figure 1 and reported in Table 2. In the month after treatment initiation, the self-transitions from 1 stage to the same stage dominated. Chances of self-transitions were lower in more severe IGA stages, with estimated probabilities of 0.60 (95% CI, 0.58-0.61) for IGA 4 to IGA 4 and 0.80 (95% CI, 0.78-0.82) for IGA 0/1 to IGA 0/1 (Figure 1). The probability of self-transitions declined as the follow-up duration increased. Probabilities of transitions of only 0.22 (95% CI, 0.20-0.23) for IGA 4 to IGA 4 and 0.54 (95% CI, 0.51-0.57) for IGA 0/1 to IGA 0/1 were identified in 1 to 12 months.

**Table 2.** Posttreatment Prognoses Across the 3 Therapy Groups and for Each Therapy Group

IGA score at baseline	Transition probability, mean (95% CI)							
	0-1 mo				1-12 mo			
	IGA 0/1	IGA 2	IGA 3	IGA 4	IGA 0/1	IGA 2	IGA 3	IGA 4
**Overall**								
IGA 0/1	0.80 (0.78-0.82)	0.12 (0.10-0.13)	0.06 (0.05-0.08)	0.02 (0.01-0.03)	0.54 (0.51-0.57)	0.26 (0.23-0.28)	0.16 (0.14-0.17)	0.05 (0.04-0.06)
IGA 2	0.18 (0.17-0.19)	0.74 (0.73-0.75)	0.06 (0.06-0.07)	0.01 (0.01-0.02)	0.34 (0.33-0.35)	0.48 (0.47-0.50)	0.14 (0.13-0.15)	0.04 (0.03-0.04)
IGA 3	0.18 (0.17-0.19)	0.13 (0.12-0.13)	0.68 (0.66-0.68)	0.02 (0.02-0.02)	0.35 (0.34-0.36)	0.24 (0.23-0.25)	0.37 (0.36-0.38)	0.04 (0.03-0.04)
IGA 4	0.19 (0.18-0.21)	0.14 (0.13-0.15)	0.07 (0.06-0.08)	0.60 (0.58-0.61)	0.36 (0.34-0.37)	0.29 (0.28-0.31)	0.14 (0.12-0.15)	0.22 (0.20-0.23)
**Traditional**								
IGA 0/1	0.83 (0.80-0.85)	0.10 (0.08-0.12)	0.05 (0.04-0.07)	0.02 (0.01-0.03)	0.61 (0.57-0.65)	0.19 (0.16-0.23)	0.15 (0.12-0.18)	0.05 (0.03-0.07)
IGA 2	0.15 (0.14-0.16)	0.78 (0.76-0.80)	0.05 (0.05-0.06)	0.01 (0.01-0.02)	0.31 (0.29-0.33)	0.54 (0.52-0.56)	0.12 (0.11-0.13)	0.04 (0.03-0.04)
IGA 3	0.15 (0.13-0.16)	0.10 (0.09-0.11)	0.74 (0.72-0.75)	0.02 (0.01-0.02)	0.30 (0.29-0.32)	0.20 (0.19-0.21)	0.47 (0.45-0.48)	0.03 (0.02-0.04)
IGA 4	0.17 (0.15-0.20)	0.12 (0.10-0.14)	0.03 (0.02-0.04)	0.67 (0.64-0.70)	0.33 (0.30-0.37)	0.23 (0.20-0.26)	0.12 (0.10-0.14)	0.31 (0.28-0.34)
**Systemic**								
IGA 0/1	0.85 (0.81-0.89)	0.05 (0.03-0.07)	0.07 (0.04-0.09)	0.04 (0.01-0.06)	0.61 (0.56-0.66)	0.18 (0.14-0.22)	0.16 (0.12-0.20)	0.05 (0.03-0.07)
IGA 2	0.25 (0.23-0.27)	0.70 (0.68-0.72)	0.04 (0.03-0.05)	0.01 (0.00-0.01)	0.38 (0.36-0.41)	0.47 (0.45-0.49)	0.12 (0.11-0.14)	0.03 (0.02-0.03)
IGA 3	0.17 (0.15-0.18)	0.12 (0.11-0.13)	0.70 (0.68-0.71)	0.02 (0.01-0.02)	0.33 (0.31-0.34)	0.24 (0.23-0.26)	0.40 (0.38-0.42)	0.03 (0.03-0.04)
IGA 4	0.16 (0.14-0.19)	0.10 (0.08-0.12)	0.06 (0.04-0.07)	0.68 (0.65-0.71)	0.30 (0.28-0.33)	0.27 (0.24-0.29)	0.14 (0.12-0.16)	0.29 (0.26-0.32)
**Biologic**								
IGA 0/1	0.73 (0.69-0.77)	0.20 (0.16-0.23)	0.08 (0.05-0.10)	0	0.41 (0.36-0.45)	0.39 (0.35-0.43)	0.16 (0.13-0.19)	0.04 (0.02-0.06)
IGA 2	0.15 (0.13-0.17)	0.73 (0.71-0.75)	0.10 (0.08-0.11)	0.02 (0.01-0.03)	0.34 (0.32-0.36)	0.43 (0.41-0.45)	0.18 (0.17-0.20)	0.05 (0.04-0.06)
IGA 3	0.23 (0.21-0.24)	0.16 (0.15-0.17)	0.59 (0.57-0.60)	0.03 (0.02-0.03)	0.42 (0.40-0.44)	0.29 (0.27-0.30)	0.25 (0.24-0.27)	0.04 (0.04-0.05)
IGA 4	0.23 (0.21-0.25)	0.18 (0.17-0.20)	0.10 (0.08-0.11)	0.49 (0.47-0.51)	0.41 (0.39-0.44)	0.34 (0.32-0.37)	0.14 (0.13-0.16)	0.10 (0.09-0.12)

Abbreviation: IGA, Investigator’s Global Assessment.

The probabilities of both improvement transition into a less severe stage and deterioration transition into a more severe stage increased as the follow-up duration increased. However, improvement transitions tended to have larger increases than deterioration transitions. The probability of improvement transition of IGA 4 to IGA 0/1 increased from 0.19 (95% CI, 0.18-0.21) in 0 to 1 month to 0.36 (95% CI, 0.34-0.37) in 1 to 12 months. While the probability of deterioration transition of IGA 0/1 to IGA 4 also increased, the increases were relatively small, from 0.02 (95% CI, 0.01-0.03) in 0 to 1 month to 0.05 (0.04-0.06) in 1 to 12 months. Similarly, larger increases for improvement transitions and smaller or nonsignificant increases for deterioration transitions were found for the transitions between IGA 4 and IGA 2, IGA 3 and IGA 0/1, and IGA 3 and IGA 2 (Figure 1 and Table 2). The exception was the transitions between IGA 0/1 and IGA 2, in which the increases for the improvement transition of IGA 2 to IGA 0/1 and the deterioration transition of IGA 0/1 to IGA 2 were similar.

The identified temporal patterns remained the same for the 3 therapies. For each therapy, self-transitions declined, improvement and deterioration transitions increased, and improvement transitions generally had larger increases than deterioration transitions as the follow-up duration increased. For biologic therapy, the probability of self-transition of IGA 4 to IGA 4 was 0.49 (95% CI, 0.47-0.51) in 0 to 1 month and declined to 0.10 (95% CI, 0.09-0.12) in 1 to 12 months. Moreover, the improvement transition probability between IGA 4 and IGA 0/1 was 0.23 (95% CI, 0.21-0.25) in 0 to 1 month, and no patients progressed from IGA 0/1 to IGA 4 in 0 to 1 month; these transition probabilities changed to 0.41 (95% CI, 0.39-0.44) and 0.04 (95% CI, 0.02-0.06), respectively, in 1 to 12 months (Table 2).

### Comparison of Posttreatment Prognoses Among Therapies

**Figure 2.**
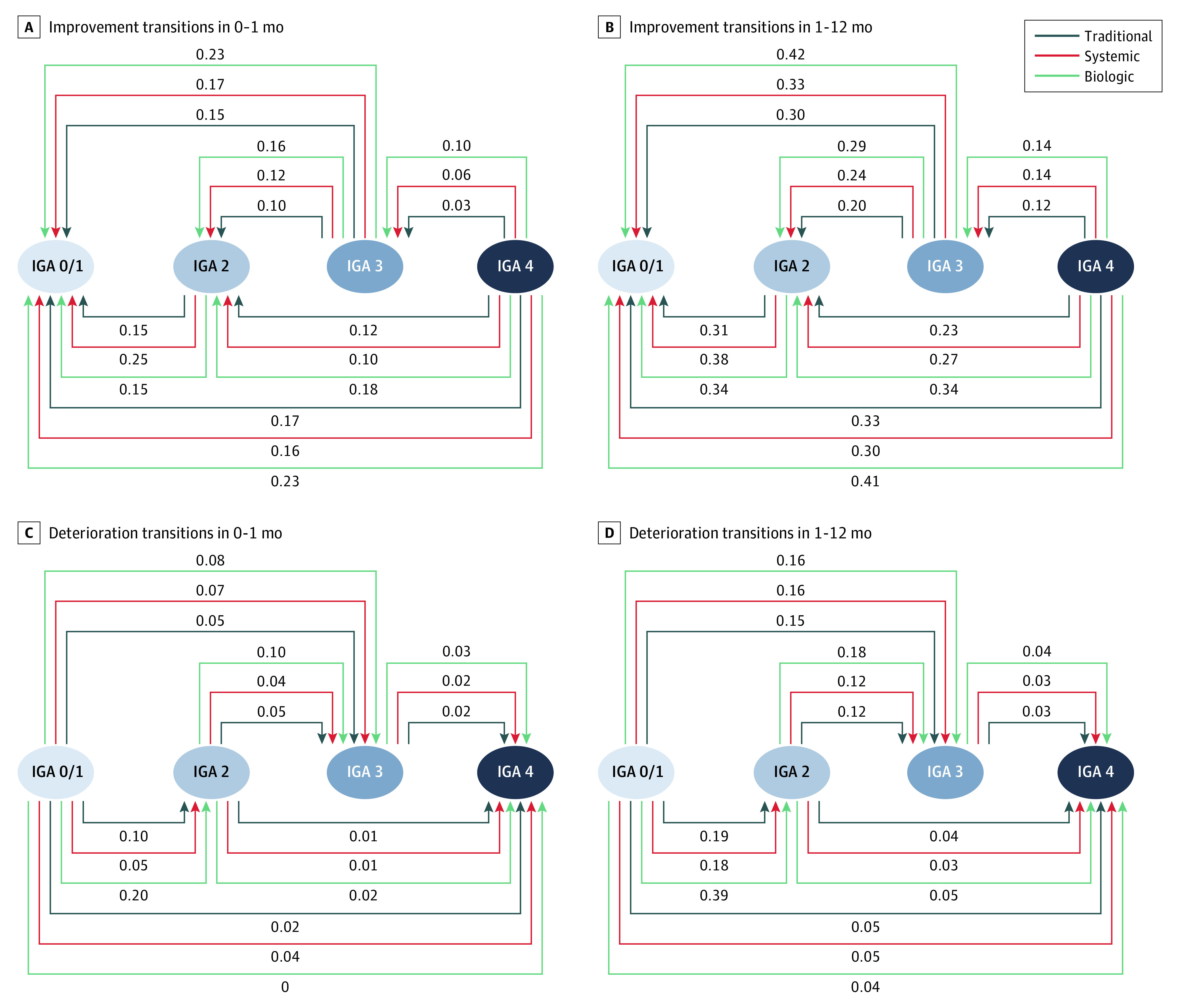
Transition Diagrams Among Investigator’s Global Assessment (IGA) Stages for Biologic, Traditional, and Systemic Therapy Data shown are probabilities of transitioning from the baseline stage to posttreatment stage in 0 to 1 months (A, C) and 1 to 12 months (B, D).

Biologic therapy was associated with more considerable improvements than the other 2 therapies in 1 month. Considering the improvement transition of IGA 4 to IGA 0/1 in 0 to 1 month, the transition probability for biologic therapy increased by 0.06 (95% CI, 0.02-0.09) compared with traditional therapy and by 0.06 (95% CI, 0.03-0.09) compared with systemic therapy (Table 3 and Figure 2). For the improvement transition of IGA 3 to IGA 0/1 in 0 to 1 month, the transition probability for biologic therapy increased by 0.08 (95% CI, 0.06-0.10) and 0.06 (95% CI, 0.04-0.08) compared with traditional therapy and systemic therapy, respectively. Finally, the transition probability of biologic therapy remained higher than the other 2 therapies for the improvement transitions of IGA 4 to IGA 2 and IGA 3 to IGA 2.

**Table 3.** Comparison of Posttreatment Prognoses for Biologic Therapy vs Traditional and Systemic Therapies

IGA score at baseline	Difference in transition probability, mean (95% CI)							
	0-1 mo				1-12 mo			
	IGA 0/1	IGA 2	IGA 3	IGA 4	IGA 0/1	IGA 2	IGA 3	IGA 4
**Biologic vs traditional therapy**								
IGA 0/1	−0.10 (−0.14 to −0.05)	0.10 (0.06 to 0.14)	0.02 (−0.01 to 0.05)	−0.02 (−0.03 to −0.01)	−0.20 (−0.26 to −0.14)	0.20 (0.14 to 0.25)	0.01 (−0.03 to 0.05)	−0.01 (−0.03 to 0.02)
IGA 2	0.00 (−0.02 to 0.02)	−0.05 (−0.07 to −0.02)	0.04 (0.03 to 0.06)	0.01 (0.00 to 0.01)	0.03 (0.00 to 0.06)	−0.11 (−0.14 to −0.08)	0.07 (0.04 to 0.09)	0.02 (0.00 to 0.03)
IGA 3	0.08 (0.06 to 0.10)	0.06 (0.04 to 0.08)	−0.15 (−0.17 to −0.13)	0.01 (0.00 to 0.01)	0.12 (0.09 to 0.14)	0.09 (0.06 to 0.11)	−0.22 (−0.24 to −0.19)	0.01 (0.00 to 0.02)
IGA 4	0.06 (0.02 to 0.09)	0.06 (0.03 to 0.09)	0.06 (0.05 to 0.08)	−0.18 (−0.22 to −0.14)	0.08 (0.04 to 0.12)	0.11 (0.07 to 0.15)	0.02 (0.00 to 0.05)	−0.21 (−0.25 to −0.18)
**Biologic vs systemic therapy**								
IGA 0/1	−0.12 (−0.17 to −0.07)	0.15 (0.11 to 0.19)	0.01 (−0.03 to 0.04)	−0.04 (−0.06 to −0.01)	−0.20 (−0.27 to −0.14)	0.21 (0.16 to 0.27)	0.00 (−0.05 to 0.05)	−0.01 (−0.04 to 0.02)
IGA 2	−0.10 (−0.12 to −0.07)	0.03 (0.01 to 0.06)	0.05 (0.04 to 0.07)	0.01 (0.00 to 0.02)	−0.05 (−0.08 to −0.02)	−0.04 (−0.07 to −0.01)	0.06 (0.04 to 0.09)	0.03 (0.01 to 0.04)
IGA 3	0.06 (0.04 to 0.08)	0.04 (0.02 to 0.06)	−0.11 (−0.14 to −0.09)	0.01 (0.00 to 0.02)	0.09 (0.07 to 0.12)	0.04 (0.02 to 0.07)	−0.15 (−0.17 to −0.12)	0.01 (0.00 to 0.02)
IGA 4	0.06 (0.03 to 0.09)	0.08 (0.06 to 0.11)	0.04 (0.02 to 0.06)	−0.19 (−0.23 to −0.15)	0.11 (0.07 to 0.14)	0.08 (0.04 to 0.11)	0.01 (−0.02 to 0.03)	−0.19 (−0.23 to −0.16)

Abbreviation: IGA, Investigator’s Global Assessment.

The probability of improvement transition of biologic therapy increased greatly after 1 month. An estimated 0.08 (95% CI, 0.04-0.12) increase in transition probability compared with traditional therapy and 0.11 (95% CI, 0.07-0.14) increase compared with systemic therapy were found for the improvement transition of IGA 4 to IGA 0/1 in 1 to 12 months (Table 3, Figure 2). Also, the transition probability of biologic therapy compared with the other 2 therapies increased substantially for the improvement transitions of IGA 4 to IGA 2 and IGA 3 to IGA 0/1 or IGA 2.

However, for the improvement transition of IGA 2 to IGA 0/1, systemic therapy was associated with small but significant increases compared with the other 2 therapies in both 0 to 1 month and 1 to 12 months (Table 2). In addition, biologic therapy was associated with considerably higher deterioration transitions of IGA 0/1 to IGA 2 and IGA 2 to IGA 3 than the other therapies (Figure 2 and Table 3).

## Discussion

We modeled the posttreatment prognosis of skin lesions using transition diagrams and estimated the transition probabilities with data from a large cohort of patients with psoriasis in China. We found that as the follow-up duration increased from 0 to 1 month to 1 to 12 months, the probability of improvement transitions into a less severe IGA stage increased, self-transitions into the same stage declined, and deterioration transition into a more severe stage increased, though with smaller magnitudes. The findings were consistent for biologic therapy, traditional therapy, and systemic therapy. Compared with traditional and systemic therapies, biologic therapy was associated with significantly increased improvement transitions starting from moderate and severe skin lesions and with deterioration transitions starting from mild skin lesions, while systemic therapy performed better than traditional or biologic therapy for mild psoriasis.

Consistent with a review^3^ summarizing the prognosis of skin lesions at 12 to 16 weeks, this cohort study identified an increasingly favorable prognosis for all 3 therapies as the follow-up duration increased until a year. The finding highlights the importance of enhancing patient adherence to therapies, especially for those who might not respond in a month after treatment initiation. Since the increase from a favorable prognosis was greater than that from an unfavorable prognosis, improved patient adherence may lead to better patient satisfaction. Nevertheless, a considerable number of patients with moderate skin lesions did not respond to any of the 3 therapies after a year of treatment, a finding suggesting that more dynamic biologic therapy strategies for patients with less severe skin lesions are needed to improve patient satisfaction.

Biologic therapy was found to be associated with improved prognosis for patients with moderate and severe skin lesions, consistent with earlier findings.^17,37,38^ The improvement transitions of IGA scores of 3 and 4 to 0/1 within 1 month were similar to previous results in 2 clinical trials^13^ with secukinumab, the primary biologic in the present study. Although the improvement transitions from IGA scores of 3 and 4 to 0/1 in 1 to 12 months were lower than the finding (around 60%) of 2 clinical trials^13^ at the end point of 12 months, this could be due to the shorter follow-up duration and more adaptable therapy strategies in the present study. Unlike the previous clinical trials, the dose and frequency of receipt of biologics in this prospective cohort study were adjusted often based on the patient’s tolerance.

Furthermore, we estimated that the transition probability associated with biologic therapy compared with the other 2 therapies increased considerably as the follow-up duration increased. Biologics provide treatment convenience to patients because of the less frequent administration compared with other therapies.^39,40^ However, due to the high out-of-pocket costs, the frequency of use of biologics in Chinese clinical practice is still low.^41,42^ Including biologics in general government-funded health care may be key to resolving this issue. Currently, only 6 biologics (secukinumab, ixekizumab, ustekinumab, etanercept, infliximab, and adalimumab) are included in the National Drug Reimbursement List in China and are primarily used for severe skin lesions resistant to systemic therapy. Our results call for efforts in advocacy and education to ensure that biologic treatment is accessible to all patients.

Transition diagrams among IGA scores presented several advantages in clinical use. First, transition probabilities among IGA scores focus on changes in skin lesions, and thus, like PASI improvement (eg, PASI 75 response, which refers to a 75% or greater reduction in PASI), they can capture the treatment needs of patients.^8,43^ However, unlike PASI improvement, the transition diagrams present not only improvements in skin lesions but also self-transitions and deteriorations, providing more complete information on the posttreatment prognoses. Second, the transition diagrams are visually understandable, which can help patients understand the risks and benefits of each therapy and build proper expectations of posttreatment prognoses. In addition, the alignment with earlier findings on advantages of biologic therapy^17,37,38^ increases confidence in using transition diagrams. These findings suggest that transition diagrams may be useful in understanding the prognosis of skin lesions in clinical practice and may help facilitate the dialogue between clinicians and patients.

Our study adds to the literature in several ways. First, although investigating the posttreatment prognosis of skin lesions is not novel, to our knowledge, this study was the first to model the posttreatment prognosis of skin lesions using transition diagrams. Transition diagrams include both favorable and unfavorable prognoses, providing more complete information on the developments of diseases. Transition diagrams are visually understandable by clinicians and patients and can help to intuitively understand both the chances and risks of different therapies. Second, this study is, to our knowledge, the first to investigate a large amount of observational data from clinical practice in China. Compared with clinical trials, observational data allow for complex treatment decisions on doses and frequency of administration of therapies, and thus, we believe they generate more clinically valid estimates. Third, our findings support the relevance of enhancing treatment adherence in managing psoriasis^44,45,46^ and adoption of biologic therapy.^39^ The findings are of relevance for China at this early phase of using biologics.^42^

### Limitations

This study has several limitations. The current study is an observational study, and while we used the matching method to reduce the measured confounding, there may exist unmeasured confounding that was not captured by the current data. A potential limitation is that patients who were treated with biologic therapy were more likely to be wealthy with more healthy diet habits and thus may have tended to have shorter time to remission. Furthermore, the follow-up duration in our study represented the natural clinical practice in China, which was considerably shorter than 1 year for most participants. Although the resulting transition diagrams are informative for understanding the posttreatment prognosis at the population level, they cannot be interpreted as the yearly transition diagram at the individual level. Finally, we considered a short-term prognosis of skin lesions after therapies, and the results might not be directly extrapolated into the long term for more than 1 year.

## Conclusions

In this cohort study modeling psoriasis prognosis in China, biologic therapy was associated with greater improvement in skin lesions in patients with moderate to severe psoriasis compared with traditional and systemic therapies in the first month and even greater improvement in the first year of treatment, which highlights the need to use biologic therapy for these patients and the importance of treatment adherence for better improvement. The findings provide insight on using transition diagrams to assess psoriasis prognosis and to communicate with patients in clinical practice.

## Notes

Supplement 1. **eAppendix 1.** Definition of Comorbidities **eFigure.** Flowchart of Study Population Inclusion and Exclusion **eAppendix 2.** Hypothesis Testing **eTable.** Results of Hypothesis Testing

Supplement 2.
Data Sharing Statement
