# Supplementary material for: Modeling Posttreatment Prognosis of Skin Lesions in Patients With Psoriasis in China
Source: JAMA Netw Open. 2023 Apr 6;6(4):e236795. doi: 10.1001/jamanetworkopen.2023.6795 (PMC10080370; doi:10.1001/jamanetworkopen.2023.6795)
Supplement: Supplement 1. — eAppendix 1. Definition of Comorbidities eFigure. Flowchart of Study Population Inclusion and Exclusion eAppendix 2. Hypothesis Testing eTable. Results of Hypothesis Testing [file jamanetwopen-e236795-s001.pdf]

Supplemental Online Content

Yang Z, Han S, Wu P, et al. Modeling posttreatment prognosis of skin lesions in patients with psoriasis in China. *JAMA Netw Open*. 2023;6(4):e236795.
doi:10.1001/jamanetworkopen.2023.6795

eAppendix 1. Definition of Comorbidities

eFigure. Flowchart of Study Population Inclusion and Exclusion

eAppendix 2. Hypothesis Testing

eTable. Results of Hypothesis Testing

This supplementary material has been provided by the authors to give readers additional information about their work.

© 2023 Yang Z et al. *JAMA Network Open*.

eAppendix 1. Definition of comorbidities

Comorbidities included in the studies are rheumatic immune diseases (e.g. psoriatic arthritis), cardio-metabolic diseases (e.g. major adverse cardiovascular events such as myocardial infarction and stroke, hypertension, diabetes, and dyslipidemia), gastrointestinal diseases (e.g. inflammatory bowel disease and hepatic disease), kidney disease, respiratory disease (e.g. chronic obstructive pulmonary disease and obstructive sleep apnea), malignancy, infection, other dermatological diseases, mood disorders, and psychiatric diseases. Any comorbidity before enrollment is included in the baseline characteristics.

© 2023 Yang Z et al. *JAMA Network Open*.

eFigure 1. Flowchart of study population inclusion and exclusion

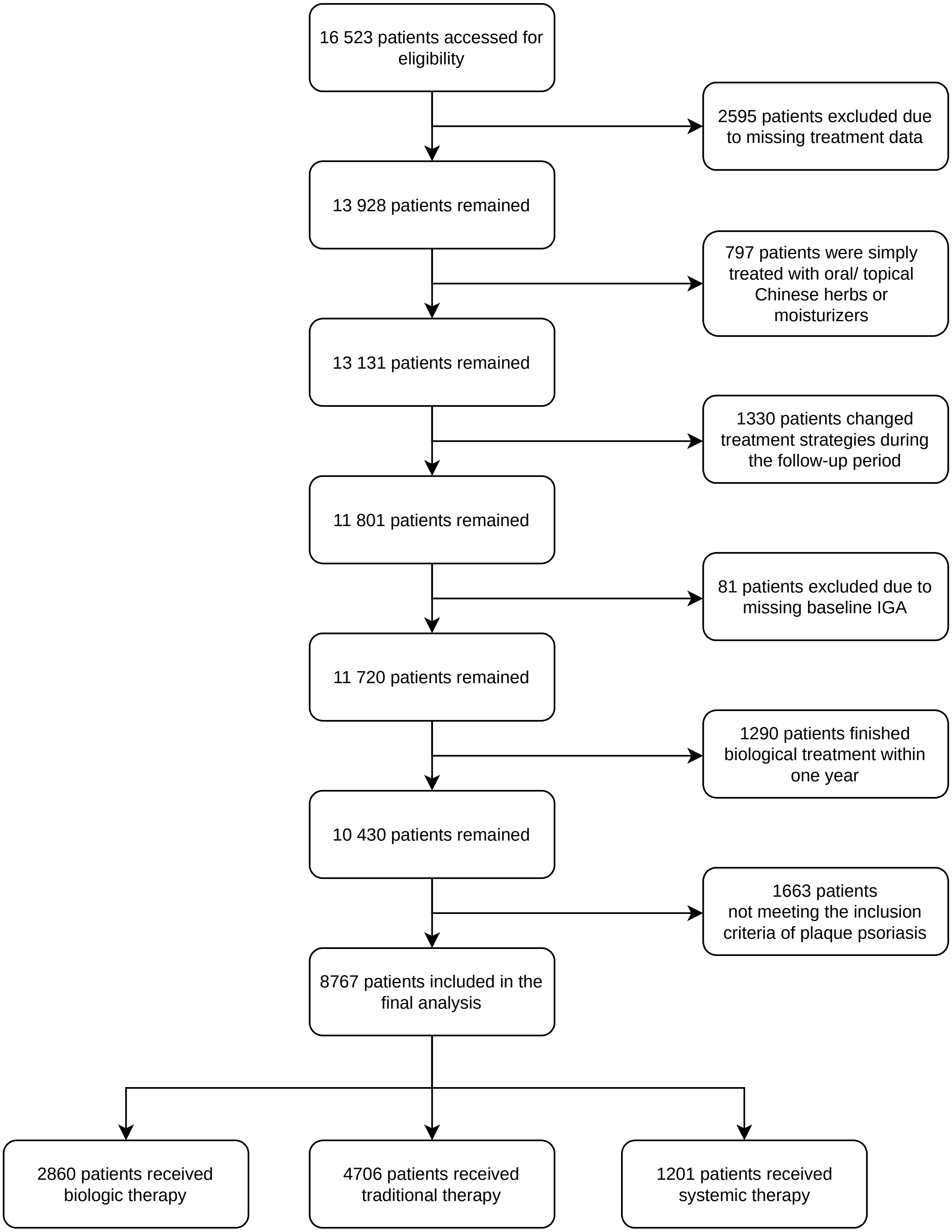

IGA, Investigator's Global Assessment

© 2023 Yang Z et al. *JAMA Network Open*.

eAppendix 2. Hypothesis testing

We performed the one-sided T-test to assess the significance of the reduction in standardized mean differences after matching, compared with these before matching. The testing procedure was described as follows:

For each pair of therapy comparison (i, j), and each covariate k, we calculated the standardized mean differences, as

$$\Delta_{ki,kj} = \frac{|\mu_{ki} - \mu_{kj}|}{\sqrt{(\sigma_{ki}^2 + \sigma_{kj}^2)/2}},$$

where μ_{ki} and μ_{kj} are the means of the covariate values for therapy groups i and i, respectively, σ_{ki} and σ_{ti} represent the standard errors respectively.

For each pair of therapy comparison (i, j), and each covariate k, we calculated the standardized mean differences before and after matching separately. For clarity, we denoted them as $\Delta_{ki,kj}^{before}$ and $\Delta_{ki,kj}^{after}$. We then calculated the t-statistics as

$$T = (\Delta_{t1,t2}^{before} - \Delta_{t1,t2}^{after})/\sqrt{2},$$

where $\sqrt{2}$ is the normalization factor.

Finally, we calculated P values with the one-sided test, with null hypothesis $H_0 : T \leq 0$ and alternative hypothesis $H_1 : T > 0$.

The results are displayed in eTable 1. Majorities of covariates had p-values significantly less than 0.0001, with (100%,28/28) for the pairs of biologic therapy and traditional therapy, (86%, 24/28) for the pairs of biologic therapy and systemic therapy, and (86%, 24/28) for the pairs of systemic therapy and traditional therapy.

© 2023 Yang Z et al. *JAMA Network Open*.

eTable 1. Results of hypothesis testing

	P-values (Balance Improvement, Before Match vs. After Match)		
	**Biologic versus Traditional**	**Biologic versus Systemic**	**Traditional versus Systemic**
**Sex**			
**Female**	<.001	.10	<.001
**Male**	<.001	.10	<.001
**Age, median, years**	<.001	<.001	<.001
**BMI, median, kg/m^2**	<.001	>.99	<.001
**Marital status**			
**Married**	<.001	<.001	<.001
**Unmarried**	<.001	<.001	<.001
**Employment**			
**Full-time**	<.001	<.001	>.99
**Others**	<.001	<.001	>.99
**Educational level**			
**College degree or higher**	<.001	<.001	<.001
**High school or lower**	<.001	<.001	<.001
**Smoking status**			
**Current everyday smoker**	<.001	<.001	<.001
**Current sometime smoker or nonsmoker**	<.001	<.001	<.001
**Psoriasis duration, median, years**	<.001	<.001	<.001
**Family history of psoriasis**			
**Positive**	<.001	<.001	.005
**Negative**	<.001	<.001	.005
**Lesions on particular areas**			
**Nail affected**	<.001	<.001	<.001
**Nail unaffected**	<.001	<.001	<.001
**Scalp affected**	<.001	<.001	<.001
**Scalp unaffected**	<.001	<.001	<.001
**Palmoplantar affected**	<.001	<.001	<.001
**Palmoplantar unaffected**	<.001	<.001	<.001
**Genital affected**	<.001	<.001	<.001
**Genital unaffected**	<.001	<.001	<.001
**Disease severity**			
**PASI, median**	<.001	<.001	<.001
**BSA, median**	<.001	.08	<.001
**DLQI, median**	<.001	<.001	<.001
**Comorbidities**			
**Positive**	<.001	<.001	<.001
**Negative**	<.001	<.001	<.001

BMI, body mass index (calculated as weight in kilograms divided by height in meters squared); BSA, body surface area; DLQI, Dermatology Life Quality Index; PASI, Psoriasis Area and Severity Index.

© 2023 Yang Z et al. *JAMA Network Open*.
