# Supplementary material for: Modeling Posttreatment Prognosis of Skin Lesions in Patients With Psoriasis in China
Source: JAMA Netw Open. 2023 Apr 6;6(4):e236795. doi: 10.1001/jamanetworkopen.2023.6795 (PMC10080370; doi:10.1001/jamanetworkopen.2023.6795)
Supplement: Supplement 2. — Data Sharing Statement [file jamanetwopen-e236795-s002.pdf]

Data Sharing Statement

Yang. Modeling Posttreatment Prognosis of Skin Lesions in Patients With Psoriasis in China. *JAMA Netw Open*. Published April 06, 2023. doi:10.1001/jamanetworkopen.2023.6795

Data
Data available: No

Additional Information
Explanation for why data not available: This study was conducted using deidentified data from the data collection platform of Psoriasis Standardized Diagnosis and Treatment Center in China. Raw data are not publicly available.
